# Oral Administration of Royal Jelly Restores Tear Secretion Capacity in Rat Blink-Suppressed Dry Eye Model by Modulating Lacrimal Gland Function

**DOI:** 10.1371/journal.pone.0106338

**Published:** 2014-09-22

**Authors:** Toshihiro Imada, Shigeru Nakamura, Naoki Kitamura, Izumi Shibuya, Kazuo Tsubota

**Affiliations:** 1 Department of Ophthalmology, Keio University School of Medicine, Tokyo, Japan; 2 Department of Veterinary Physiology, Faculty of Agriculture, Tottori University, Tottori, Japan; Save Sight Institute, Australia

## Abstract

Tears are secreted from the lacrimal gland (LG), a dysfunction in which induces dry eye, resulting in ocular discomfort and visual impairment. Honey bee products are used as a nutritional source in daily life and medicine; however, little is known about their effects on dry eye. The aim of the present study was to investigate the effects of honey bee products on tear secretion capacity in dry eye. We selected raw honey, propolis, royal jelly (RJ), pollen, or larva from commercially available honey bee products. Tear secretion capacity was evaluated following the oral administration of each honey bee product in a rat blink-suppressed dry eye model. Changes in tear secretion, LG ATP content, and LG mitochondrial levels were measured. RJ restored the tear secretion capacity and decrease in LG ATP content and mitochondrial levels to the largest extent. Royal jelly can be used as a preventative intervention for dry eye by managing tear secretion capacity in the LG.

## Introduction

The ocular surface, comprising the cornea and conjunctiva, is covered by a thin layer of an aqueous tear film secreted from the lacrimal gland (LG). The role of tear fluid is to provide a proper environment to maintain homeostasis for the ocular surface [1]. The tear film acts as the first defense system against environmental microbes, desiccation, and foreign bodies and also smooths the refractive surface of the transparent cornea [2]. Dry eye disease is a multifactorial disorder characterized by the status of the tear film, results in ocular discomfort and visual impairment [3], and has become a major public health issue in industrial societies that have many of the risk factors for this disease, including the use of digital devices (computers, tablets, and smart phones) [4]. The incidence of dry eye was previously showed to be particularly high in workers that stare at the screens of technological devices [5], [6]. We previously demonstrated that a chronic reduction in tear production was induced by extended computer use in both human and animal studies, and suggested that LG hypofunction may be a critical mechanism in digital device-induced dry eye [7]. The function of the LG also has been shown to decrease with aging, a known potent risk factor for dry eye [8], [9]. Therefore, the discovery and development of novel preventative interventions that could maintain healthy LG function may have considerable clinical implications. However, temporal tear replacement therapy, the frequent application of artificial tear eye drops, has long been used in the basis management of dry eye [10].

Beekeeping products have been deeply rooted in the lives of different people and cultures worldwide for thousands of years as a nutritional source and medicine. The main products of beekeeping are honey, royal jelly, propolis, pollen, and bee larva produced or collected by Apidae. These products exhibit a wide range of biological effects, including antibacterial [11], [12], antiviral [13], anti-inflammatory [14], [15], and/or antiallergenic actions [16], [17]. Honey eye drops have been used in corneal wound therapy as a traditional medicine [18] in the ophthalmic field and propolis was shown to protect against neuronal damage in the retina [19]. However little is known about the effects of honey bee products on tear secretion capacity in dry eye.

In the present study, we assessed the potential usefulness of orally applied honey bee products as a preventative intervention in dry eye associated with the excessive use of digital devices. We used a blink-suppressed dry eye model: persistent strain by swing treatment in combination with exposure to an evaporative environment, which induces disordered tear dynamics, decreased blink frequency, and LG dysfunction. This dry eye model was established based on the concept that the long-lasting use of technological devices is associated with a marked reduction in the frequency of blinking while gazing at the screen and awkward postures in a dry environment [7].

## Materials and Methods

### Animals

Female 8-weeks-old Sprague-Dawley rats (CLEA Japan, Japan) were used in this study. All animals were quarantined and acclimatized for 1 week prior to the experiments under the following general conditions: room temperature of 23±2°C, relative humidity of 60±10%, alternating 12-hour light-dark cycle (8 AM to 8 PM), and water and food *ad libitum*. All animals were used according to the Association of Research and Vision in Ophthalmology (ARVO) statement for the Use of Animals in Ophthalmic and Vision Research. The protocol for this study was approved by the Ethics Committee on Animal Research of the Keio University School of Medicine (Approval No. 11008-2).

### Honey bee products

Raw honey, an ethanolic extract of propolis, the lyophilized powder of RJ, pollen granules, and the lyophilized powder of larva were used. The detailed information of each honey bee products used in this study is described in Table S1. All honey bee products were supplied by Yamada Bee Company, Inc. (Okayama, Japan).

### Protein secretion from isolated LGs

Rats were euthanized by excess pentobarbital administration and their LG were rapidly dissected. LG digested by collagenase type 3 (Worthington, USA) was incubated in saline solution (140 mM NaCl, 5 mM KCl, 2 mM CaCl_2_, 1 mM MgCl_2_, 10 mM HEPES, 10 mM dextrose [pH 7.4]) with each honey bee product. Carbachol (CCH), a cholinergic stimulus, was used as a positive control. The protein concentration in the medium was measured using the Bradford reagent (Sigma-Aldrich, USA) with bovine serum albumin (BSA) as the standard. The protein secretion rate was calculated as a percentage of that the before stimulation. 5 rats were used in each group.

### Rat blink-suppressed dry eye model

The model and methodology used to simulate VDT has been described previously [7], [20], [21]. In brief, a series of treatments were performed under dry conditions, with a room temperature of 23±2°C, relative humidity of 25±5%, and constant air flow at 2 to 4 m/s produced by an electric fan. Each rat was placed on a swing for 7.5 hours per day between 9 AM and 5 PM. A photograph of the rat blink-suppressed model was shown in figure S1. This series of treatment, procedure to simulate VDT in rat, was repeated for 5 days for screening of honey bee products and 10days for the RJ evaluation. Each honey bee product was dissolved in distilled water at 240 mg/ml (honey), 40 mg/ml (pollen, larva, and propolis), and 60 mg/ml (RJ) and was repeatedly administered orally each 1 ml to rats once a day. Distilled water was used as vehicle control. To screen honey bee products at the effective dose for health benefits, the dosage of each honey bee product was chosen by reference to previous reports [14], [22], [23]. Lacrimal function was evaluated on day 11. For each experiment using rat blink-suppressed dry eye model, 6 to 18 rats were used in each group.

#### Tear secretion

We used a modified Schirmer test on rat eyes to measurement tear fluid secretion under topical anesthesia by 0.4% oxybuprocaine hydrochloride solution (Santen Pharmaceutical, Japan) [24]. A phenol red thread (Zone-Quick; Showa Yakuhin kako, Japan) was placed on the temporal side of the upper eyelid margin for 1 minute. The length of the moistened area from the edge was measured within 1 mm.

#### Corneal fluorescein staining

Changes in the corneal surface were determined by the application of a fluorescein solution under a blue-free barrier filter [25], and corneal staining of the area was graded according to previously described criteria [20].

#### Histopathological examination

The fixed LG in 10% formalin was embedded in paraffin and sectioned. Sections were subjected to HE staining or immunostaining. The acinar cell density of each section was determined by quantifying the nuclear number in three randomly selected areas (2500 µm^2^).

Vesicle-associated membrane protein 8 (VAMP8) was immnostained to evaluate the occupied pattern of secretory vesicles (SVs) in the acinar cells of specimens. VAMP8 was previously shown to be enriched on the membranes of zymogen granules [26]. The primary antibody used for immunostaining was a rabbit monoclonal antibody against VAMP8 (Abcam, UK). Nuclear staining was performed by treating specimens with hematoxylin.

#### Mitochondrial function

The homogenized LG was examined to measure ATP levels and mitochondrial content. ATP levels were determined according to the instructions for the ATP Bioluminescence Assay Kit CLS 2 (Roche Molecular Biochemicals, Germany). Mitochondrial content was measured by using the fluorescence dye, Mitotracker green FM (Molecular Probes, USA) and Hoechst 33342 (Dojin Chemical, Japan). These measurements were performed on a Synergy 4 plate reader (Biotek Company, USA). The mitochondrial membrane potential of the LG was visualized by staining with Mitotracker red (Invitrogen), a membrane potential-dependent fluorescence dye.

#### Western blot

Total protein extracts from the LG were separated by polyacrylamide gel electrophoresis, transferred to PVDF membranes using a dry blotting system (V20-SDB, SCIE-PLAS, UK), and incubated with antibodies. Immunolabeled proteins were detected using Pierce Western blotting Substrate Plus (Thermo SCIENTIFIC, Germany) and a Lumino-image analyzer (LAS-4000, FujiFilm, Japan). All bands were normalized to β-actin. The primary antibodies used for Western blotting were as follows; AMP-activated protein kinase [AMPK (Cell Signaling, Japan), Phospho-AMPK (Cell Signaling)], and β-actin (Sigma Aldrich). 6rats were used in each group.

### Intracellular calcium ion concentration ([Ca^2+^]i ) measurements

The LG digested by collagenase type 3 was filtered through a 100 µm nylon mesh (Cell Strainer, BD Biosciences, Japan) to isolate LG acinar cells. Acinar cells were loaded with fura-2/AM (Invitrogen, USA), the fluorescent Ca^2+^ indicator, and transferred to round coverslips. Coverslips were then mounted on a chamber fixed on the stage of an inverted fluorescence microscope (IX71, Olympus, Japan). Acinar cells were continuously perfused with diverse experimental solutions through polyethylene tubes connected to a peristaltic pump (Minipulse 3, Gilson, USA) at flow rate of 0.8 ml per minutes.

Royal jelly (100, 300, and 500 µg/ml), CCH (10 µM; Tokyo Kasei Kougyo, Japan), and 10-hydroxy-2-decenoic acid (500 µg/ml; 10-HDE, Cayman, USA) were diluted to the desired concentrations with saline solution and used as stimulants. AG1478 (10 µM; Wako, Japan), Atropine sulfate (1 µM; Nacalai, Japan), U73122 (1 µM; Sigma, Japan), and cyclopiazonic acid (10 µM; CPA, Sigma, Japan) were appropriately prepared in saline solution before use. Changes in [Ca^2+^]i were measured by dual excitation microfluorometry using a digital image analyzer (Aqua Cosmos/Ratio, Hamamatsu Photonics, Japan). The fluorescence signal was detected using UV objective lens (UApo 20×3/340, Olympus), and the emission passing through a band pass filter (500±10 nm) was detected by a cooled CCD camera (ORCAER, Hamamatsu Photonics, Japan). Data acquisition was performed at a sampling frequency of 0.2 Hz throughout the experiments using Aqua Cosmos software (Hamamatsu Photonics). The fluorescence intensity of each pixel was measured and digitalized at the excitation wavelengths of 340 and 380 nm by the analyzing software (Aqua Cosmos). For each experiment, 10 to 53 acinar cells were used in each group.

### Statistical analysis

The Student’s *t*-test was used to compare the two groups and Dunnett’s test was used for multiple comparisons. Differences between the measured variables were considered significant if the resultant *P* value was 0.05 or less.

## Results

### Effects of honey bee products on tear secretion capacity

**Figure 1:**
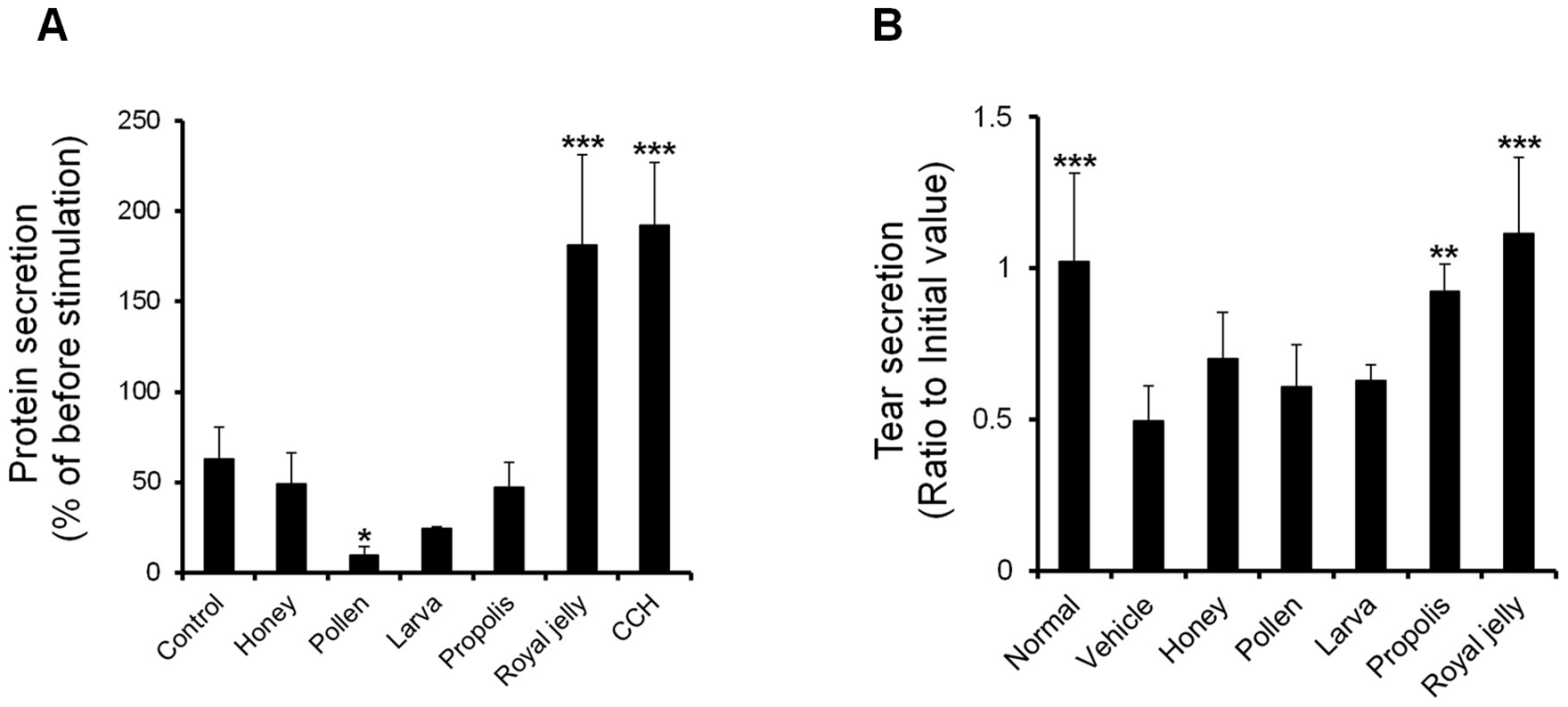
Evaluation of various honey bee products. A: Effect of various honey bee products on tear secretion in a rat blink-suppressed dry eye model. The oral administration doses of honey, pollen, larva, propolis, and RJ were 1200, 200, 200, 200, and 300 mg/kg, respectively (n = 5 rats). B: Protein secretion rate from normal LG after stimulation by various honey bee products. The LG was stimulated with 500 µg/ml of each honey bee product (n = 5 LG). All data represent the mean ± SD. *P<0.05, **P<0.01, ***P<0.001 versus the vehicle.

Figure 1 shows the effects of the screened honey bee products on tear secretion capacity. In the dry eye model, which corresponded to that of our previous study [7], tear secretion was significantly less in the vehicle group than in the normal group. The prevention of a reduction in tear secretion was significantly more prominent in the RJ and propolis group than in the vehicle group (Fig 1A). In the other products (honey, pollen, and larva) and vehicle, significant decreases were observed (Fig 1A).

Protein secretion from LG acinar cells were significantly increased by the RJ and CCH stimulation. The stimulation of protein secretion was not observed with the other products (Fig 1B).

### Evaluation of the rat blink-suppressed dry eye model

**Figure 2:**
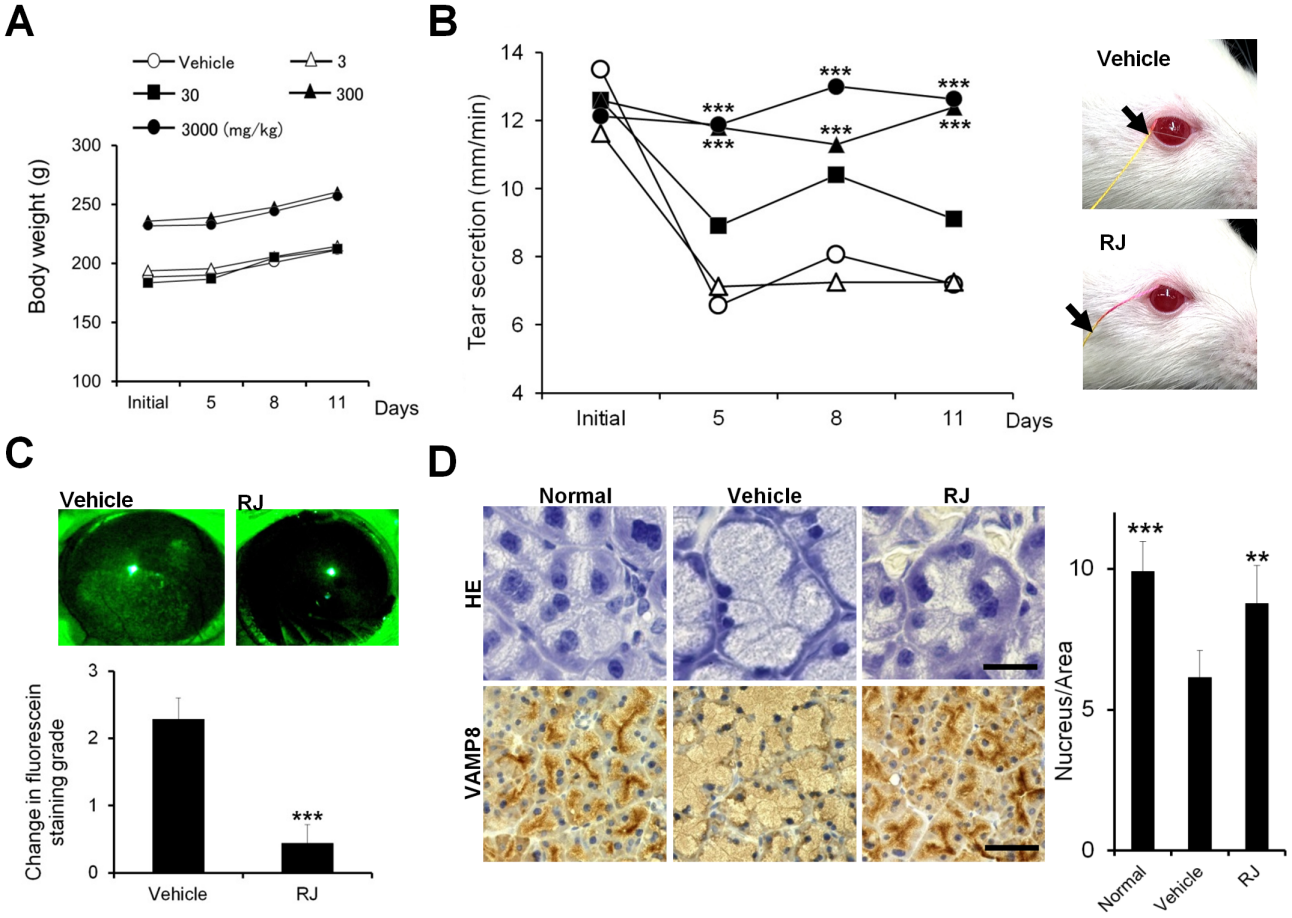
Royal jelly restored tear secretion in a rat blink-suppressed dry eye model. A: Changes in body weight. B: Changes in tear secretion (Left). (n = 10 rats) Representative photographs of tear secretion patterns measured by a cotton thread (Right). The arrow shows the wetted length by tear secretion. C: Effect of RJ on corneal surface damage. Changes in the grading score (lower, n = 9 rats), Typical pattern of staining (Upper). Punctate staining appeared in the whole area of cornea surface with the vehicle treatment. D: Histopathological changes in the LG. HE staining (Left upper). VAMP8 immunostaining (Left lower). Scale bar, 20 µm (HE) or 50 µm (VAMP8). Acinar cell size (Right, n = 6 LG). All data represent the mean ± SD. **P<0.01, ***P<0.001 versus the vehicle.

A significant difference was not observed in body weight gain among any dose of RJ and vehicle groups (Fig 2A). Tear secretion was significantly less from 5 days to 11 days than the initial value in the vehicle group.

RJ dose-dependently suppressed the reduction in tear secretion. This suppression was significantly stronger with 300 and 3000 mg/kg RJ than with the vehicle at all time points throughout the experiment (Fig2B, left). Figure 2B right shows representative photographs of tear secretion patterns measured by the modified Schirmer test. The wetted length on the thread showed by a red discolored area was shorter in vehicle eyes than in RJ eyes.

We performed corneal epithelial fluorescein staining on 300 mg/kg RJ at day 11 to investigate whether the effects of RJ on tear secretion altered corneal epithelial damage in the dry eye condition. The grade of corneal epithelial fluorescein staining was significantly higher in the vehicle than the initial value. The intake of RJ maintained the graded score at the same level as the initial value and the graded score was significantly less than that of the vehicle. Representative SPK patterns restored by RJ are shown in Figure 2C.

### Alteration in LG morphology

Figure 2D shows a representative microphotograph of the LG stained by HE (upper) and secretory vesicle-occupied pattern by VAMP8 immunostaining (lower) in the dry eye condition. In the vehicle group, the cytoplasm of acinar cells was occupied by more enlarged acini with expanded cytoplasm than that of the normal group. Aciner cell density was significant reduced in the vehicle than in the normal value of the dry eye condition. RJ maintained the aciner cell density as the same level as the normal value, and the value was significantly higher than that of vehicle (Fig. 2D, bar chart).

VAMP8 was primary localized near the apical membrane in the normal state, while it was distributed diffusely in the entire cytoplasm of acinar cells in the vehicle group. VAMP8 was localized to the proximity of the apical membranes in the RJ group and its distribution was identical to that found in the normal state. These results suggest that acinar cells were enlarged by the accumulation of secretory vesicles and returned to the normal state by the intake of RJ in this dry eye model.

### Alteration in mitochondrial function in the LG

**Figure 3:**
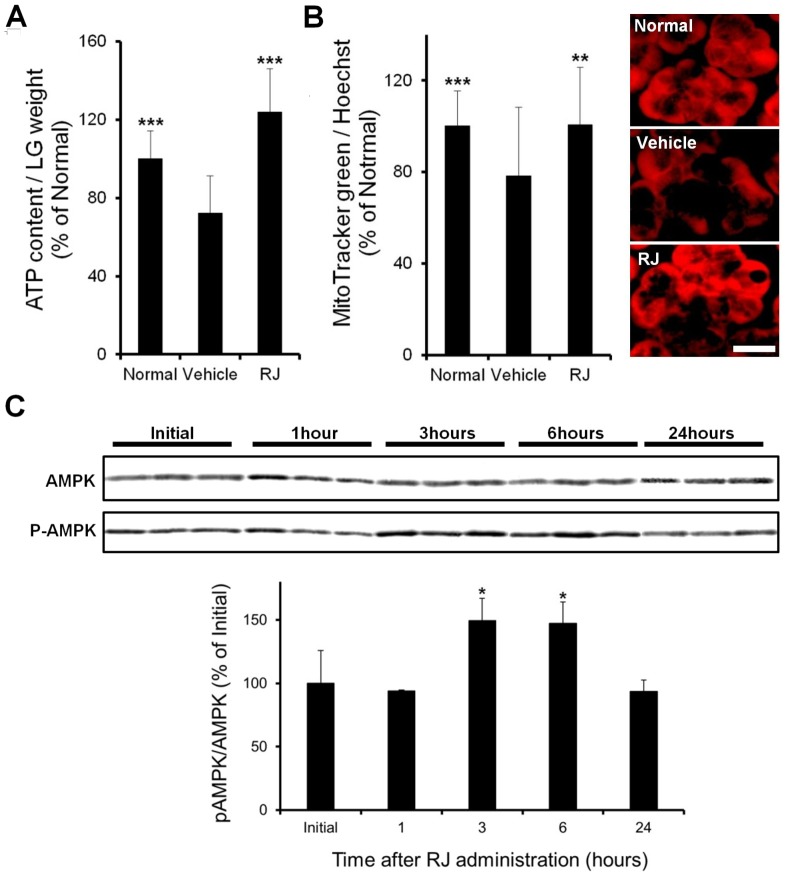
Effect of royal jelly on lacrimal gland function. A: ATP levels. B: Mitochondria content (Left). Imaging of the mitochondrial membrane potential (Right). Scale bar, 20 µm. (n = 12–18 LG) C: AMPK activity in a normal lacrimal gland after the oral administration of RJ (n = 6 LG). All data represent the mean ± SD. * *P*<0.05, **P<0.01, ***P<0.001, versus the vehicle (ATP and mitochondria) or initial (AMPK).

ATP levels were significantly lower in the vehicle group than in the normal group. The intake of RJ significantly restored the decrease in ATP levels in the vehicle group (Fig 3A). Mitochondrial content was significantly lower in the vehicle group than in the normal group. Mitochondrial content was restored significantly more in the RJ group than in the vehicle group (Fig 3B). Figure 3B right shows a representative image of mitochondrial activity detected by staining with a membrane potential-dependent fluorescein probe. The accumulation of fluorescein probe in acinar cells was less in the vehicle group than in the normal group. The accumulation of the probe was observed in the entire cytoplasm of acinar cells in the RJ group. These results suggest that mitochondrial activity in the LG was restored by the intake of RJ in the dry eye condition.

### Effect of RJ on AMPK phosphorylation in the LG

The oral intake of RJ induced a significant increase in AMPK phosphorylation after 3 and 6 hours, and this increase returned to initial levels after 24 hours (Figure 3C).

### Effect of RJ on LG [Ca^2+^]i mobilization

**Figure 4:**
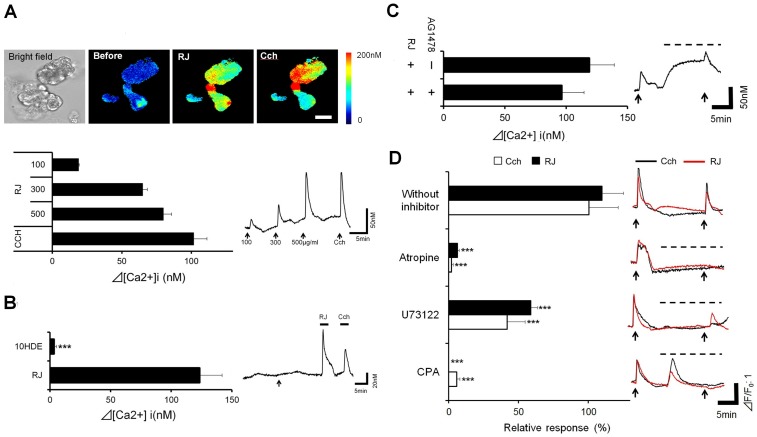
Effect of royal jelly on lacrimal gland acinar [Ca^2+^]i mobilization. A: Changes in [Ca^2+^]i in normal lacrimal acinar cells. Pseudo colored images of [Ca^2+^]i (Upper). Scale bar, 50 µm. The bar graph shows the summarized data of the amplitudes of [Ca^2+^]i responses. (n = 13 acini) B: Effect of 10-HDE (Left, n = 14 acini). The typical response to 10HDE (Right). C: Effect of AG1478 (n = 10 acini). D: Effect of muscarinic acetylcholine pathway inhibitors (Left, n = 22–53 acini). Responses to the stimulation with or without inhibitors (Right). The arrow indicates the time at which RJ or CCH was applied to the cells. Dotted line over the trace indicated the presence of each inhibitor. Relative responses were calculated as a percentage of the decrease in the RJ-induced [Ca^2+^]i response with the application of an inhibitor relative to the stimulation seen with RJ alone in each acinus. All data represent the mean ± SE. ***P<0.001 versus RJ (Fig 4C) or without an inhibitor (Fig 4D).

RJ dose-dependently induced an increase in [Ca^2+^]i (Fig 4A). To investigate the calcium signaling pathway involved in this elevation, the effects of possible factors found in RJ were examined [27], [28], [29]. An elevation in [Ca^2+^]i was not detected in 10-HDE (Fig 4B). No significant change was observed in the [Ca^2+^]i elevation by RJ with or without AG1478, an epidermal growth factor receptor (EGFR) antagonist (Fig 4C). The effects of muscarinic acetylcholine pathway inhibitors on the [Ca^2+^]i elevation by RJ were shown in Figure 4D. The acetylcholine receptor antagonist atropine abolished the [Ca^2+^]i elevation. U73122 and CPA, potent inhibitors of PLC or ER-Ca^2+^-ATPase respectively, significantly suppressed the [Ca^2+^]i elevation by RJ. No significant differences were observed between the inhibitory effects of these agents on the responses to RJ and CCH.

The RJ-induced [Ca^2+^]i elevation was compared to that of raw RJ material, in order to eliminate the possibility that the activity was due to preparation process from raw material. No difference in the [Ca^2+^]i elevation was observed between the raw RJ and RJ (Fig S3). These result demonstrated that [Ca^2+^]i elevation by RJ was via active ingredients formed by Apis.

## Discussion

To verify the potential nutritional benefits of commercially available honey bee products in the prevention of dry eye, we screened the effects of each product on tear secretion capacity in this study.

Protein secretion from the LG, a critical function for tear secretion, was only evoked by RJ and the decrease in tear secretion *in vivo* in the dry eye model was restored by RJ and propolis at a dosage sufficient for human health benefits. In addition, the intake of RJ suppressed corneal epithelial damage, which is considered as one of the clinical manifestations changes on the ocular surface in dry eye. These results indicate that RJ restored dry eye symptoms by directly acting on the LG and may represent a very potent nutritional treatment for the prevention of dry eye compared with the honey bee products screened.

The formation of tear fluid by the LG is thought to be achieved by a combination of transepithelial water flow generated by an osmotic gradient and protein secretion from intracellular vesicles by exocytotic release in acinar cells [30].

The activation of multiple water channels and ion transporters were previously shown to be involved in the facilitation of a transepithelial osmotic gradient [31], [32]. The process of secretory vesicle-mediated exocytosis comprises a chain of complex mechanisms involving intracellular trafficking and fusion with the plasma membrane to release their contents into the extracellular space [33]. These secretory processes require ATP as an energy source to produce protein rich aqueous fluid from LG cells while tearing [34], [35]. We previously demonstrated that the stressful condition used in this dry eye model, an immobilized rat on a swing board in the presence of a continuous desiccated air blower, causes a decrease in tear secretion capacity accompanied an increased volume of secretory vesicles and loss of the intracellular cell structure, which indicates a dysfunction in the secretory system [7]. Exposure to stress is known to exacerbate the energy status of various organs, resulting in the loss of mitochondrial function [36], [37], [38]. In the present study, we showed a decrease in ATP levels and mitochondria content in the LG, in addition to lacrimal dysfunction. Thus, our results suggest that the reduction in the energy status in the LG is critical in the impairment of tear secretion capacity in the dry eye condition

Most cellular ATP is generated from mitochondrial oxidative phosphorylation. To maintain cellular ATP levels, [Ca^2+^]i levels play a critical role in the control of ATP synthesis by activating oxidative phosphorylation-related enzymes in mitochondria [39]. Adenosine monophosphate-activated protein kinase (AMPK), a key regulator of mitochondrial biogenesis and energy production pathways, is activated by Ca^2+^/calmodulin-dependent protein kinase kinase-β in response to an increase in the cytosolic-free calcium levels [40]. Our results demonstrated that the oral administration of RJ enhanced tear secretion capacity associated with an increased level of ATP, mitochondria, and phosphorylation of AMPK in the LG. In addition, stimulation with RJ on LG acinar cells evoked [Ca^2+^]i in a dose-dependent manner. However the mobilization of [Ca^2+^]i in LG acinar cells is believed to directly stimulate tear fluid secretion by activating ion transporters and exocytosis, and the elevated period and level in tear secretion stimulated by the intake of RJ in the normal rat was insufficient to compensate for decreased tear secretion in the dry eye condition (Fig S2).

Several substances that could potentially promote to maintain cellular ATP levels via increase in cytosolic Ca^2+^ are found in RJ. Acetylcholine, a major neurotransmitter involved in tear secretion, was found of 1 mg/g in RJ [27] and increases intracellular Ca^2+^ levels by activating muscarinic receptors. 10-hydroxy-2-decenoic acid is a unique fatty acid found in RJ that induces the entry of extracellular Ca^2+^ by activating Ca^2+^ -permeable nonselective cation channels [28]. Royalactin, a specific protein drives queen development in honeybees, acts through EGFR to increase [Ca^2+^]i [29].

Our results showed that 10-HDE did not stimulate [Ca^2+^]i, and a muscarinic receptor inhibitor, but not EGFR abolished RJ-induced [Ca^2+^]i mobilization. Furthermore, RJ-induced [Ca^2+^]i mobilization was suppressed by inhibitors of PLC and ER-Ca^2+^-ATPase, which are essential enzymes involved in the muscarinic signal transduction pathway [41]. These results demonstrated, for the first time, to the best of our knowledge, that RJ mobilizes [Ca^2+^]i through the muscarinic signal transduction pathway and suggest that preserving the energy status via this pathway is a potential mechanism by which RJ restores tear secretion capacity. To further identify and characterize the active components of RJ on tear secretion capacity, a separation and purification technique such as bioassay-guided fractionation with chemical structure analysis is needed.

In conclusion, this study is the first to demonstrate that orally administrated RJ restores tear secretion capacity in dry eye. This effect was assumed to be associated with the LG energy status through modulation of the calcium signaling pathway stimulated by RJ. Since the safety of RJ is well-known, further clinical studies on the prevention or treatment by RJ with a focus on information technology-associated dry eye will contribute to establish new therapeutic interventions for this disease.

## Supporting Information

Figure S1 **Photograph of the rat blink-suppressed dry eye model.** Electric fan (A), swing (B). In addition to being placed on the swing, the rats were exposed to constant low humidity air flow aimed at the face, produced by an electric fan.
(TIF)

Figure S2 **Changes in tear secretion after RJ was administered orally to normal rats.** The dose of the oral administration was 3000 mg/kg RJ. Data represent the mean ± SD for 6 rats. *P* values versus the vehicle.
(TIF)

Figure S3 **Changes in [Ca^2+^]i elevation by raw RJ.** The acini were stimulated by 500 µg/ml of raw RJ. (n = 18–24 acini) Data represent the mean ± SE.
(TIF)

Table S1 **Information of each honey bee product.**
(TIF)

Methods S1 **Supporting methods.**
(DOCX)
