# Supplementary figures and images for: Oral Administration of Royal Jelly Restores Tear Secretion Capacity in Rat Blink-Suppressed Dry Eye Model by Modulating Lacrimal Gland Function
Source: PLoS One. 2014 Sep 22;9(9):e106338. doi: 10.1371/journal.pone.0106338 (PMC4171376; doi:10.1371/journal.pone.0106338)

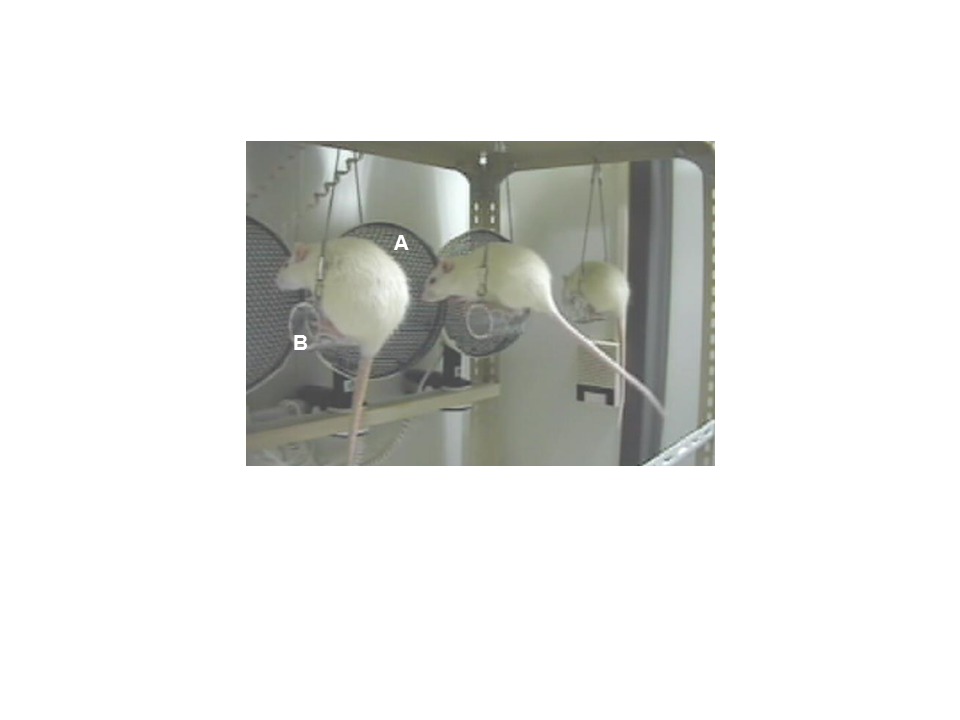

Supplement: Figure S1 — Photograph of the rat blink-suppressed dry eye model. Electric fan (A), swing (B). In addition to being placed on the swing, the rats were exposed to constant low humidity air flow aimed at the face, produced by an electric fan. (TIF) [file pone.0106338.s001.tif]

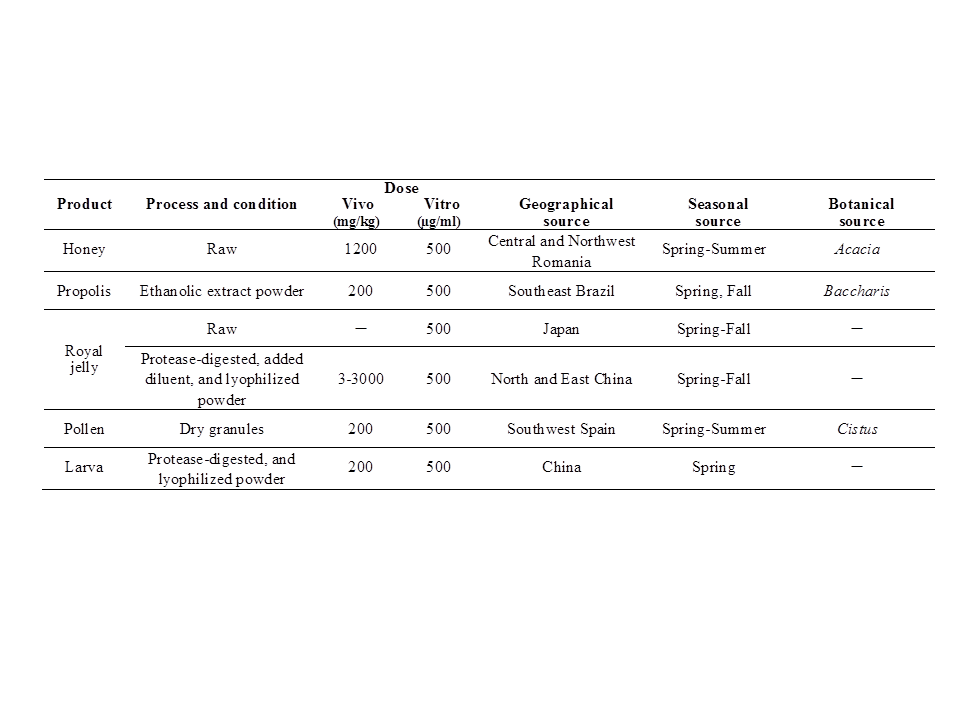

Supplement: Table S1 — Information of each honey bee product. (TIF) [file pone.0106338.s004.tif]
