# Supplementary material for: Oral Administration of Royal Jelly Restores Tear Secretion Capacity in Rat Blink-Suppressed Dry Eye Model by Modulating Lacrimal Gland Function
Source: PLoS One. 2014 Sep 22;9(9):e106338. doi: 10.1371/journal.pone.0106338 (PMC4171376; doi:10.1371/journal.pone.0106338)
Supplement: Methods S1 — Supporting methods. (DOCX) [file pone.0106338.s005.docx]

**Supporting materials and methods**

**Change in tear secretion capacity by the oral administration of RJ.**

Each rat received 3000mg/kg RJ or water (vehicle) orally. The measurement of tear secretion capacity was performed 5 minutes before RJ was administered orally and 5, 10, and 20 minutes later. Tear secretion was measured as described in the Materials and methods.

**Changes in [Ca^2+^]i elevation by raw RJ.**

Raw RJ was supplied by Yamada Bee Company, Inc. (Okayama, Japan). The [Ca^2+^]i was measured as described in the Materials and methods.
